# Prognostic Impact of Mesenteric Lymph Node Status on Digestive Resection Specimens During Cytoreductive Surgery for Ovarian Peritoneal Metastases

**DOI:** 10.1245/s10434-023-14405-3

**Published:** 2023-10-22

**Authors:** Ali Channawi, Florin-Catalin Pop, Charif Khaled, Maria Galdon Gomez, Michel Moreau, Laura Polastro, Isabelle Veys, Gabriel Liberale

**Affiliations:** 1 Department of Surgery, Institut Jules Bordet (Hopitaux Universitaires de Bruxelles [HUB]), Université Libre de Bruxelles, Brussels, Belgium; 2 Department of Pathology, Institut Jules Bordet (Hopitaux Universitaires de Bruxelles [HUB]), Université Libre de Bruxelles, Brussels, Belgium; 3 Statistics Department, Institut Jules Bordet (Hopitaux Universitaires de Bruxelles [HUB]), Université Libre de Bruxelles, Brussels, Belgium; 4 Département of Medical Oncology, Institut Jules Bordet (Hopitaux Universitaires de Bruxelles [HUB]), Université Libre de Bruxelles, Brussels, Belgium

## Abstract

**Background:**

The most common mode of ovarian cancer (OC) spread is intraperitoneal dissemination, with the peritoneum as the primary site of metastasis. Cytoreductive surgery (CRS) with chemotherapy is the primary treatment. When necessary, a digestive resection can be performed, but the role of mesenteric lymph nodes (MLNs) in advanced OC remains unclear, and its significance in treatment and follow-up evaluation remains to be determined. This study aimed to evaluate the prevalence of MLN involvement in patients who underwent digestive resection for OC peritoneal metastases (PM) and to investigate its potential prognostic value.

**Methods:**

This retrospective, descriptive study included patients who underwent CRS with curative intent for OC with PM between 1 January 2007 and 31 December 2020. The study assessed MLN status and other clinicopathologic features to determine their prognostic value in relation to overall survival (OS) and progression-free survival (PFS).

**Results:**

The study enrolled 159 women with advanced OC, 77 (48.4%) of whom had a digestive resection. For 61.1% of the patients who underwent digestive resection, MLNs were examined and found to be positive in 56.8%. No statistically significant associations were found between MLN status and OS (*p* = 0.497) or PFS ((*p* = 0.659).

**Conclusions:**

In anatomopathologic studies, MLNs are not systematically investigated but are frequently involved. In the current study, no statistically significant associations were found between MLN status and OS or PFS. Further prospective studies with a systematic and standardized approach should be performed to confirm these findings.

Ovarian cancer (OC) is the eighth most prevalent cancer among women worldwide and one of the leading causes of cancer-related mortality because clinical manifestations are uncommon and diagnosis is frequently delayed. In 2020, 70% of OC in Belgium was diagnosed as International Federation of Gynecology and Obstetrics (FIGO) stages III and IV disease.^1^

Cytoreductive surgery (CRS) and carboplatin-paclitaxel-based chemotherapy are the pillars of the treatment for primary OC with peritoneal metastases (PM).^2^ The completeness of CRS is one of the most significant prognostic factors.^3^ This includes total hysterectomy with bilateral adnexectomy, omentectomy, and resection of all visible macroscopic PM (with resection of associated organs), depending on the location and size of the tumor implants.

The classification for the completeness of cytoreduction (CC) is based on the extent of disease remaining after surgery. Cytoreduction is determined to be complete (CC-0) when there is no macroscopic residual disease, optimal (CC-1) when the residual disease is less than 2.5 mm in size, and suboptimal (CC-2) when the residual disease is between 2.5 and 2.5 cm in size.^4^ Therefore, in the case of digestive involvement, including the small bowel and/or the colon and/or the colorectal hinge, digestive resections must be performed, and mesenteric lymph nodes (MLNs) may be affected.^5–11^

The involvement of MLNs in digestive cancers such as colorectal cancer is associated with increased risk of locoregional recurrence, distant metastasis, and poorer overall survival (OS).^12,13^ This MLN involvement also is taken into consideration when clinicians are deciding on adjuvant treatment and follow-up intensity in colorectal cancer.^14^

The role of MLN involvement in advanced OC remains unclear, and its significance in treatment and follow-up evaluation remains to be determined. A few retrospective studies have examined the prognostic significance of MLN status in digestive tract resection specimens obtained during cytoreduction of ovarian PM.^5–11^ Contradictory results have been reported regarding the clinical outcome for patients related to MLN status, with some studies indicating a significant correlation between MLN status and OS^10,11^ and others finding no significant correlation.^8,9^

The primary aim of this study was to evaluate the prevalence of MLN involvement in patients who underwent digestive resection for OC PM and to investigate the potential prognostic value of MLN involvement in terms of OS and PFS. The secondary goal was to evaluate the associations between clinicopathologic factors and MLN status.

## Methods

### Study Design

This retrospective, monocentric, descriptive study was conducted at the Jules Bordet Institute. The Jules Bordet Institute is accredited by the Organization of European Cancer Institutes (OECI) and by the European Society of Gynecological Oncology (ESGO) for the treatment of locally advanced OC.

The study included patients who underwent CRS with curative intent for OC PM between 1 January 2007 and 31 December 2020. Some patients underwent primary surgery, whereas others received neoadjuvant (NACT) due to a peritoneal cancer index (PCI) higher than 20, the need to do more than one or two gastrointestinal resections, or both.

On 24 January 2023, the Ethics Committee of the Jules Bordet Institute (accreditation no. OM011) approved the study (internal no. CE3590).

### Data Collection

Patient data and information were extracted from the hospital's electronic medical record system (Oribase). The data for each patient included age, body mass index (BMI), NACT and adjuvant chemotherapy (ACT), extent of peritoneal disease expressed by the PCI, pathologic FIGO (pFIGO) stage (post-NACT and postoperative), breast cancer gene (BRCA) mutations, postoperative complications according to the Clavien-Dindo classification, type of bowel resection, operative time, blood loss, the CC, histologic type, grade, tumor differentiation, extent of bowel invasion, number and status of resected MLNs, and pelvic and para-aortic lymph node involvement.

### Inclusion and Exclusion Criteria

The FIGO classification was used to determine the stage of the disease based on its dissemination during the initial treatment. The study included only patients who underwent CC-0 or CC-1 CRS for advanced OC (stage III or IV). The study excluded patients with an initial early-stage OC (stage I or II) and/or who underwent incomplete surgery (CC-2 or CC-3) and/or were treated for recurrent OC.

### Data Treatment

In addition to the number of metastatic lymph nodes and their status, the study evaluated the logarithmic odds of positive lymph nodes (LODDS) and the lymph node ratio (LNR). The LODDS was calculated using the natural logarithm of the ratio between the probability of a lymph node being positive and the probability of a lymph node being negative. To calculate the LNR, the number of metastatic lymph nodes was divided by the total number of lymph nodes examined.

### Analytical Statistics

The statistical analysis included a descriptive analysis of the study population, with characteristics reported as mean ± standard deviation or percentage according to the type of variable. The frequency and percentage of categorical and nominal variables are reported. Means, medians, and interquartile ranges are reported for continuous variables.

The relationship between the clinicopathologic variables and the presence of MLN was analyzed using the chi-square test. The relationship between the variables studied, OS, and PFS was analyzed using the Kaplan–Meier method (KM). The statistical analyses were performed using SAS version 9.4 (SAS Institute Inc, Cary, NC).

## Results

### Patient Selection and Characteristics

**Fig. 1:**
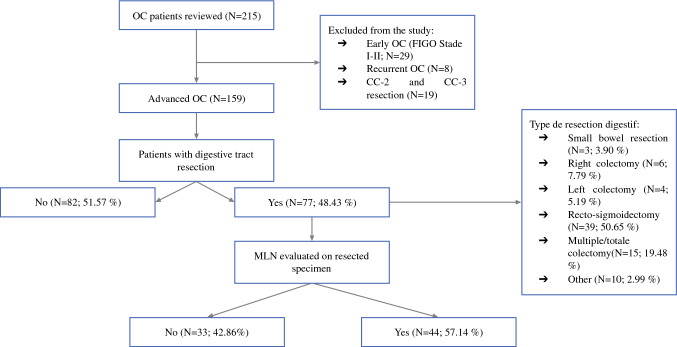
Flowchart of patients included in this study

During the period between January 2007 and December 2020, 215 patients underwent CRS for OC PM at the Jules Bordet. The study excluded 29 patients who had an early-stage OC (stage I or II), 19 patients who had undergone incomplete cytoreduction (CC-2 or CC-3), and 8 patients who were hospitalized for recurrent OC (Fig. 1).

Finally, the study included 159 women with a diagnosis of advanced OC who were treated with CC-0 or CC-1 CRS. Of these 159 patients, 130 (81.76%) had stage III OC, whereas 29 (18.24%) had stage IV OC. The mean age of the patients was 57.5 years (median, 59.71 years), and the mean PCI was 11.90 (median, 9).

According to the pathologic data, serous OC was the most common histologic type of OC, involving 143 patients (89.9%), whereas 4 patients (2.52%) had endometroid OC and 12 patients (7.55%) had other types of OC. The tumor in 118 patients (74.2%) was grade 3. Of the 159 patients, 126 (79.25%) received NACT followed by interval CRS, whereas 33 (20.75%) underwent upfront surgery.

The surgical data are shown in Table 1. Almost all the patients (98.74%) underwent CC-0, and two patients had CC-1. Pelvic lymphadenectomy was performed for 81 patients (50.9%) and lombo-aortic lymphadenectomy for 79 patients (49.7%).

**Table 1:** MLN status according to the type of digestive resection, FIGO stage, and pathology characteristics

	Patients with negative MLN *n* (%)	Patients with positive MLN *n* (%)	p Value
Type of bowel resection			
Right colectomy	1 (2.27)	1 (2.27)	NA
Left colectomy	2 (4.55)	1 (2.27)	
Recto-sigmoidectomy	10 (22.73)	10 (22.73)	
Multiple/total colectomy	3 (6.82)	7 (15.91)	
Small bowel resection	1 (2.27)	0 (0)	
Other	2 (4.55)	6 (13.64)	
BRCA mutations			
Yes	2	4	0.0753
No	9	7	
FIGO stage			
III	16 (36.36)	21 (47.73)	0.58
IVA	2 (66.7)	1 (33.3)	
IVB	1 (25.0)	3 (75.0)	
Histologic type			
Serous	16 (36.36)	21 (47.73)	0.46
Endometroid	1 (2.27)	0 (0)	
Other	2 (4.55)	4 (9.09)	
Pathologic tumor grade			
G1	4 (9.09)	4 (9.09)	0.706
G2	1 (2.27)	3 (6.82)	
G3	14 (31.82)	18 (40.91)	
Depth of bowel infiltration			
No infiltration	6 (13.64)	1 (2.27)	
Serosa	8 (18.18)	12 (27.27)	
Subserosa	0 (0)	4 (9.09)	0.133
Muscularis propria	2 (4.55)	3 (6.82)	
Mucosa	1 (2.27)	2 (4.55)	
Not specified in the anatomopathologic report	2 (4.55)	3 (6.82)	
Pelvic lymph node status			
Negative	7 (31.82)	2 (9.09)	0.421
Positive	8 (36.36)	5 (22.73)	
Lombo-aortic lymph node status			
Negative	6 (30)	2 (10)	0.067
Positive	4 (20)	8 (40)	

*MLN*, mesenteric lymph nodes; *FIGO*, International Federation of Gynecology and Obstetrics

Of the 159 patients, 77 (48.43%) had a digestive resection and 82 (51.57%) did not. Recto-sigmoidectomy was the most common digestive resection, performed for 56% of the patients with digestive resections.

### Mesenteric Lymph Node Status

In 44 (57.14%) of the 77 patients who underwent digestive resection, MLNs were examined, whereas in the remaining 33 operative specimens (42.86%), no MLNs were reported in the pathology report. Of the 44 patients, 25 (56.8%) had positive MLNs, whereas the remaining 19 patients (43.2%) had negative MLNs. The mean number of MLNs collected was 18, and of these, a mean of 4.9 MLNs were positive. Of the 44 reported patients with an MLN status, 22 had BRCA gene testing.

### Univariate Analysis Between MLN Status and Clinicopathologic Variables

The study found no association between MLN involvement and BRCA mutations (*p* = 0.075), FIGO stage (*p* = 0.58), histologic type (*p* = 0.460), depth of bowel infiltration (*p* = 0.133), or pelvic or lombo-aortic lymph node status (Table 1).

### Survival Analysis

A survival analysis was performed to determine the relationship between MLN involvement and OS and PFS. The median duration of the patient follow-up evaluation was 50.7 months.

**Fig. 2:**
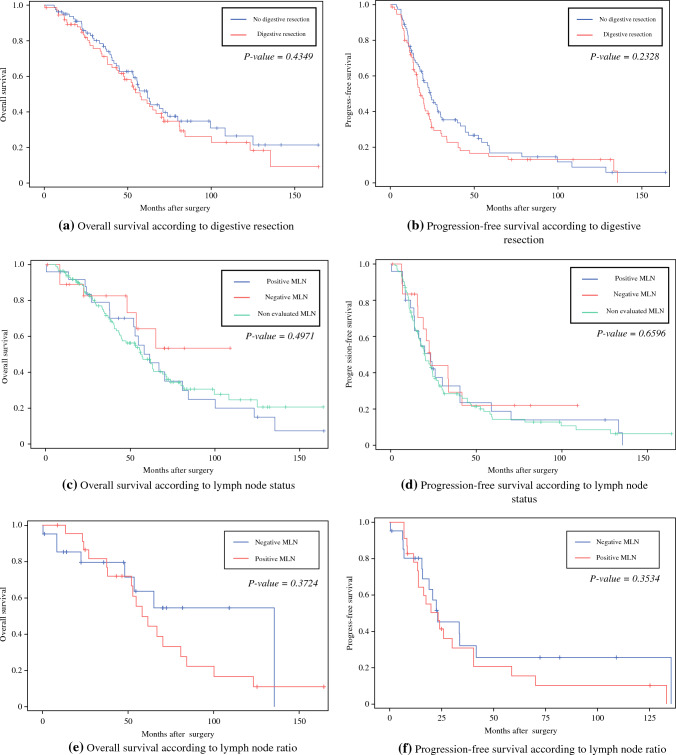
Kaplan–Meier survival curves for digestive resection and mesenteric lymph node involvement

Kaplan–Meier survival curves showed no statistically significant difference (*p* = 0.49), but we observed that the patients with MLN involvement had a slightly shorter OS than the patients without MLN involvement (Fig. 2). The estimated median survival for the patients with MLN involvement was 53.5 months versus 67.9 months for the patients without MLN involvement. Moreover, the difference in PFS between the two groups was not statistically significant (*p* = 0.65). However, it is worth noting that the patients with MLN involvement tended to have a shorter PFS (median PFS, 25.9 months) than the patients without MLN involvement (median PFS, 39 months). For LNR and LODDS, the results showed no significant correlation with OS (*p* = 0.37 and 0.35, respectively) or PFS (*p* = 0.35 and 0.38, respectively).

## Discussion

This study investigated the prevalence of MLN involvement in patients who underwent digestive resection for OC PM. Only 61.11% of the patients with digestive resections had a reported pathologic status for MLN.

The study also analyzed associations between MLN involvement and outcomes for the patients with locally advanced OC. No statistically significant associations between MLN involvement and either OS (*p* = 0.497) or PFS (*p* = 0.353) were observed in this population. In addition, none of the clinicopathologic factors studied were associated with MLN involvement (Table 1).

Macroscopic complete (CC-0) or near complete (CC-1) cytoreduction is the goal of surgery because it has been associated with improved OS and PFS.^15,16^ A review of 14 studies published since 2003 found that patients with no visible tumor after CRS had a higher OS rate than patients with persistent disease.^6^ More recently, we reported the impact of conservative and aggressive surgical attitudes on patient outcomes.^17^ For the patients in the aggressive surgical attitude group, OS was improved (*p* = 0.05), and PFS demonstrated a trend toward improvement (*p* = 0.29). To achieve complete macroscopic CRS for patients with digestive involvement, digestive resections must be performed. For these patients, the MLN status may influence patient outcomes.^5–11^

Our first objective concerned the rate of MLNs reported in the pathology report of the patients who underwent a digestive resection. Only 61% of the MLNs on resected digestive specimens were evaluated. In the literature, this rate varies from 33% to 100% (Table 2).^5–11^ In colorectal cancers, the number of MLNs found on the operative specimen is a quality indicator of the surgical resection, and the MLN status is a major prognostic factor. Therefore, MLNs on the operative specimen are systematically evaluated. Conversely, no recommendation exists for patients with OC.

**Table 2:** Previous reports regarding digestive resection and MLN involvement in ovarian cancer

	No. of digestive resections	Types of digestive resection included	NACT *n* (%)	MLN evaluated on resected specimen	Positive MLN *n* (%)	OS & PFS
O’Hanlan KA et al.^5^ *Gynecol Oncol.* (1995)	100	Any	N/S	33 (33)	24 (70)	Yes (2 years)
Salani R et al.^6^ *Ann Surg Oncol.* (2007)	53	Recto-sigmoidectomy	N/S	39 (73)	31 (79.4)	N/S
Baiocchi G et al.^7^ *J Surg Oncol.* (2011)	50	Any	N/S	41 (82)	29 (70.7)	N/S
Gouy S et al.^8^ *Eur J Surg Oncol.* (2012)	52	Any	34 (65)	52 (100)	19 (37)	No (3 years)
Gallotta V et al.^9^ Ann Surg Oncol. 2014	148	Recto-sigmoidectomy	37 (25)	102 (68.9)	48 (47.0 )	No (2 years)
Berretta R et al.^10^ *Arch Gynecol Obstet.* (2018)	83	Any	16 (19.3)	76 (91.6)	43 (51.8)	Yes (20 months)
Tanaka K et al.^11^ *Ann Surg Oncol.* (2021)	27	Recto-sigmoidectomy	N/S for digestive resection for 85 OC patients 39 (45.8)	27 (100)	14 (52)	Yes
Our study (2023)	77	Any	61 (79)	44 (61.11)	25 (56.)	No

*MLN*, mesenteric lymph node; *NAC*, neoadjuvant chemotherapy; *OS*, overall survival; *PFS*, progression-free survival; *N/S*, not specified

In the future, to improve understanding of the role of MLN status for patients with locally advanced OC who underwent CRS, MLNs should be systematically identified on the operative specimen and reported on the pathology report. A more systematic approach would be to develop a standardized pathologic report for MLNs in patients with OC. Such a report could include detailed information on the number and location of affected MLNs as well as any relevant pathologic characteristics such as depth of bowel infiltration, metastatic spread, or lymph node size. For example, in the current series, the depth of bowel infiltration was not specified in nine pathologic reports (11.69%). The lymph node size was not specified in any pathologic report. This type of standardized reporting would increase the uniformity of MLN evaluation across institutions and allow more accurate comparisons between studies.

The second objective of this study was to evaluate the association between MLN status and outcomes (OS and PFS). Systematic lymphadenectomy for pelvic and lombo-aortic lymph nodes has been a matter of debate in the past due to mixed results in previous retrospective studies. However, current randomized clinical trials, such as the Lymphadenectomy in Ovarian Neoplasm (LION) study, do not support the practice of systematic lymphadenectomy for locally advanced OC.^18,19^ The results of the LION study indicate that this procedure may not significantly improve OS (*p* = 0.65) or PFS (*p* = 0.29) for patients with OC and is associated with a higher incidence of postoperative complications (*p* = 0.01).

In the current study, a survival analysis was performed to determine the relationship between MLN involvement and OS/PFS. Because previous studies on gastric, pancreatic, and papillary thyroid cancer as well as a previous study on MLN involvement in OC have suggested that LNR and LODDS are more accurate prognostic indicators than the number of positive lymph nodes,^10,20–27^ a survival analysis also was performed with them. However, no statistically significant association with OS or PFS was found, even when LNR and LODDS were considered. Some previous studies have reported that MLN involvement in OC affects OS and/or PFS, whereas other studies have reached conclusions similar to ours, reporting no correlation between MLN involvement and outcomes (Table 2).^5–11^

This situation has several possible explanations because previous studies shared the same shortcomings. First, the sample size of this study might have been too small to detect a statistically significant association. Only 77 patients underwent digestive resection, and only 44 specimens had MLNs studied. Previous studies had larger samples, such as the 83-patient study by Berreta et al.^10^ and the 148-patient study by Gallotta et al.^9^ (Table 2). However, it should be noted that these studies had varying inclusion criteria and methods, and that the types of digestive resections and MLN dissections performed were not the same.

Second, as previously mentioned, our study population may have differed in various ways from those in other research, such as treatment history. An aspect that may have influenced the results of our study is NACT. In our study, 79% of the patients who underwent a digestive resection had NACT, which is a significantly higher percentage than in other studies. For instance, Gallota et al.^9^ reported that only 25% of their patients received NACT, whereas Berrata et al.^10^<AQ3> reported a rate of only 19.3%. The only study with a similar percentage was that of Gouy et al.,^8^ at 65%, which reported similar results with no statistically significant correlation of pelvic and/or lombo-aortic lymph node involvement with PFS and OS. Although NACT does not seem to have an impact on OS, studies suggest that it may reduce tumor size and allow for more radical surgery.^28^ The use of NACT could have reduced the rate of nodal spread and possibly modified the results of the current series, thus explaining the different results between Gouy et al.,^8^ our study, and other studies. However, this is also debatable because other reports suggest that NACT does not affect the rate of nodal involvement in OC.^8,26–30^

Third, our study may have defined MLN involvement and digestive resection differently than previous studies. The digestive resections included in our analysis may not be directly comparable with those of previous studies because the extent of bowel resection and lymphadenectomy performed varied considerably. For example, the studies by Salani et al.^6^ and Gallotta et al.^9^ included only patients who underwent recto-sigmoidectomy, whereas different types of digestive resections were included in our study. This may have influenced the results because the patterns of lymphatic spread could be different depending on the location of the tumor implant. The different surgical techniques and extents of resection could potentially affect the rate of MLN involvement and survival outcomes. Future studies with larger samples and more standardized surgical techniques may help to further define the relationship between MLN involvement and survival of patients with OC who have undergone different types of digestive resections.

Finally, the third objective of this study was to evaluate whether some clinicopathologic variables were associated with MLN status.

First, this study found no significant association between MLN status and FIGO stage (*p* = 0.58), histologic type (*p* = 0.46), or tumor grade (*p* = 0.7). A comparison of these findings with those in the literature showed that none of the previous studies provided significant evidence supporting a correlation between the previously mentioned clinicopathologic variables (Table 3).^5–11^

**Table 3:** Previous reports regarding clinicopathologic features and MLN involvement in ovarian cancer

	FIGO stage	Histologic type	Pathologic tumor grade	Depth of bowel infiltration	PLN	LALN
O’Hanlan KA et al.^5^ *Gynecol Oncol.* (1995)	N/S	N/S	No (*p* = 0.14)	No (*p* = 0.08)	N/S	N/S
Salani R et al.^6^ *Ann Surg Oncol.* (2007)	N/S	No (*p* = NS)	N/S	Yes (*p* < 0.01)	N/S	No (*p* = 0.025)
Baiocchi G et al.^7^ *J Surg Oncol.* (2011)	N/S	No (*p* = 0.068)	No (*p* = 0.73)	Yes (*p* = 0.036)	No (*p* = 0.11)	Yes (*p* = 0.002)
Gouy S et al.^8^ *Eur J Surg Oncol.* (2012)	N/S	No	No	No	No	No
Gallotta V et al.^9^ *Ann Surg Oncol.* (2014)	N/S	No (*p* = 0.49)	No (*p* = 0.37)	Yes (*p* = 0.026)	No (*p* = 0.72)	No (p = 0.71)
Berretta R et al.^10^ *Arch Gynecol Obstet.* (2018)	N/S	N/S	N/S	N/S	Yes (*p* = 0.012)	Yes (*p* = 0.006)
Tanaka K et al.^11^ *Ann Surg Oncol.* (2021)	No (*p* = 0.236)	No (*p* = 0.999)	N/S	N/S	N/S	N/S
Our study (2023)	No (*p* = 0.984)	No (*p* = 0.460)	No (*p* = 0.706)	No (*p* = 0.133)	No (*p* = 0.421)	No (***p*** = 0.067)

*MLN*, mesenteric lymph node; *FIGO*, International Federation of Gynecology and Obstetrics; *PLN*, pelvic lymph node; *LALN*, lombo-aortic lymph node; *N/S*, not specified; *NS*, not significant

Second, this study did not identify a significant association between MLN status and the extent of bowel infiltration (*p* = 0.13). This finding contrasts with previous studies, such as those by Salani et al.,^6^ Baiocchi et al.,^7^ and Gallotta et al.,^9^ which reported a significant association between the depth of infiltration and MLN involvement (*p* < 0.01, 0.036, and 0.026, respectively). However, our results align with the findings of O’Hanlan et al.^5^ and Gouy et al.,^8^ both of whom also reported no significant correlation (Table 3). As previously mentioned, one potential explanation for the discrepancy could be the higher percentage of NACT, which would have reduced tumor and bowel infiltration.

Third, previous studies have shown that BRCA1 and BRCA2 mutations are associated with higher lymph node metastasis in OC.^28,29^ Our study showed no statistically significant association between BRCA mutations and MLN status (*p* = 0.075). This difference with the literature could be explained by the small sample because only 22 patients with BRCA mutations and MLN status were studied.

Finally, this study did not find a significant association of MLN involvement with pelvic (*p* = 0.42) and lombo-aortic lymph (*p* = 0.067) nodes. Previous results in the literature were mixed because this finding is consistent with the results of Salani et al.,^6^ Gouy et al.,^8^ and Gallotta et al.,^9^ whereas Baiocchi et al.^7^ and Berretta et al.^10^ reported a significant association of MLN involvement with pelvic and lombo-aortic lymph nodes (*p* = 0.002, 0.012, and 0.006, respectively; Table 3).

The main weaknesses of this study were its retrospective design, the small size of the study population, and the non-systematic reporting of the MLN status in pathologic reports. Future prospective studies with larger samples and a more systematic approach may provide more conclusive evidence of the association between MLN involvement and outcomes (OS and PFS) for patients with locally advanced OC. By addressing these limitations and exploring new avenues for research, we can continue to improve our understanding of the role of MLN involvement in OC and ultimately improve patient outcomes.

Clinical research efforts should continue to determine prognostic factors such as lymphatic routes of metastatic dissemination and biomarkers to improve OC treatment.^30,31^

## Conclusion

The results of this study highlight the lack of pathologic information on MLNs reported in pathology reports of locally advanced OC. Among patients with reported MLN information, 56% present with LN involvement. Although MLN involvement does not appear to be significantly related to OS and DFS, additional research is necessary for a full understanding of the mechanisms behind MLN metastasis in locally advanced OC. Therefore, a more systematic approach to MLN evaluation is needed. This would entail the development of a standard pathologic report for MLNs.

Our findings suggest that additional research into the physiopathology and clinical relevance of MLNs in OC is necessary. As our understanding of disease progression improves, we can expect to develop more effective treatment strategies and improve the prognosis for OC patients.
